# Longitudinal changes in quality of life and psychosocial problems of primary school children in a deprived urban neighborhood over the course of a school-based integrated approach

**DOI:** 10.1007/s00787-021-01853-z

**Published:** 2021-09-12

**Authors:** L. K. Elsenburg, H. Galenkamp, M. E. Abrahamse, J. Harting

**Affiliations:** Department of Public Health, Amsterdam UMC, University of Amsterdam, Amsterdam Public Health Research Institute, Amsterdam, The Netherlands

**Keywords:** Integrated approach, Deprived urban neighborhood, Quality of life, Psychosocial problems, Children, Primary school

## Abstract

The municipality of Amsterdam implemented a 2-year school-based integrated approach in schools in a deprived neighborhood. The integrated approach targeted the domains of education, health and poverty and the children’s school, neighborhood and home environment by involving various agencies and actors. In this study, changes in children’s quality of life and psychosocial problems over the course of the integrated approach were examined and evaluated. A dynamic cohort design was used. At five measurement occasions (T1–T5) during 2 years, children from four consecutive grades in five schools filled out a questionnaire (total *n* = 614). In children between 7 and 13 years, quality of life was measured with the KIDSCREEN-10. In children between 9 and 13 years, psychosocial problems were measured with the Strengths and Difficulties Questionnaire. Generalized estimating equations were applied. Time, sex, age, socio-economic status, ethnic background, grade, and school were included as independent variables. Quality of life was higher from the first follow-up during the approach (T2) until the end of the approach (T4) compared to at the start of the approach (T1). At T5, several months after the approach ended, scores returned back to baseline. Likewise, a reduction in children’s psychosocial problems was detected at the end of the approach (T4) compared to at the start of the approach (T1). However, both before and after that time point, no improvements were detected. This study shows that integrated approaches can be beneficial for children’s quality of life and psychosocial health, but continued investments may be needed to maintain established improvements.

*Trial registration* NTR6571 (NL6395), August 4 2017 retrospectively registered.

## Introduction

Childhood poverty is associated with developmental delay, lower cognitive performance, lower academic achievement, more psychosocial problems, and lower quality of life [1–5]. In addition, children attending a school with a large share of children from low socio-economic status (SES) have lower school achievement, more behavioral problems, and lower well-being than children from high SES schools [6]. Children living in neighborhoods with high poverty rates have lower academic skills at the start of their school career and lower high school graduation rates than children in neighborhoods with low poverty rates [7, 8]. Problems in childhood in these different domains, i.e., poverty, health, behavioral, social, and emotional development, education, academic achievement, and cognitive development, are interconnected and related to lower socio-economic status and quality of life in adulthood [9–11].

The municipality of Amsterdam recognized the multitude of problems that children, families, and schools in deprived neighborhoods face and the consequences this may bear for children’s educational trajectory and academic achievement [12]. Therefore, the municipality implemented a 2-year school-based integrated approach at schools in deprived neighborhoods. This approach aimed to improve children’s academic achievement by focusing on their education, their health, and reducing their burden of poverty [12]. In this integrated approach, various agencies and actors, e.g., school staff, policy officers, and social workers, had to collaborate to target the multitude of domains and environments in which these children experience problems. Integrated approaches are expected to be more effective in improving student outcomes than interventions focusing on single risk factors [13]. As schools are central in children’s lives, and are embedded within communities, they are considered an excellent place to roll out these integrated approaches [13, 14].

Previous school-based integrated approaches have been related to improved socio-emotional health and academic outcomes. Multifaceted approaches focusing on factors such as social support, socio-emotional health, academic development, and financial support were shown to be related to improvements in children’s socio-emotional functioning [15, 16] and academic outcomes [17]. A previous whole-school approach, which focused on improving teaching methods and positive behavior, was related to improved behavioral functioning and better academic outcomes [18]. An integrated approach, in which the theoretical frameworks and practical components of two interventions were married together, was related to a larger improvement in children’s behavior and socio-emotional health than the single interventions were [19].

In the integrated approach of the municipality of Amsterdam, participating schools received a subsidy that they could spend on self-chosen initiatives targeted at either education, health, or poverty. Schools spend the subsidy on, for example, socio-emotional health interventions, remedial teachers, new teaching methods, and broad education classes [20]. The municipality also implemented initiatives at the schools, such as a weekly consultation hour of a social worker. The process evaluation of the integrated approach showed that improving socio-emotional health was a major area of focus for the schools, and that implemented socio-emotional health initiatives were generally regarded as successful by school principals [20]. Examples are interventions focusing on stimulating positive behavior, improving the school climate, and increasing children’s resilience. The process evaluation also showed the feasibility of the implementation of this school-based integrated approach in primary schools in a deprived urban neighborhood. Connection between initiatives targeting different domains inside the school was shown to be crucial for successful implementation [20]. It made coordination of the initiatives easier and helped the school principals to safeguard the overall goal of the initiatives. Establishing this connection was shown to be aided by hired, well-trusted third parties who were able to connect agencies and actors.

The current study aimed to examine whether children’s quality of life improved and children’s psychosocial problems reduced over the course of this integrated approach and whether these improvements were sustained after the approach ended. It also evaluated whether changes in children’s quality of life and psychosocial problems were likely to be attributable to the implementation of the approach. This was done by interpreting the results of this study in light of the outcomes of the process evaluation of the implementation of the approach, which evaluated whether the program functioned as an integrated approach and why [20]. To our knowledge, this is the first study to investigate children’s socio-emotional health over the course of an integrated approach in which multiple agencies, actors, and sectors collaborated to improve outcomes in multiple domains and environments. As poverty, health, and academic achievement are interrelated, targeting all these domains will be important to establish improvements in these domains and truly establish change for children living in poverty, or in areas with high poverty rates [11, 21, 22]. Compared to at the start of the approach, we expected to identify improvements in quality of life and psychosocial problems over the course of and after the approach. However, we did expect these improvements to decrease after the approach, due to the financial support for some initiatives coming to an end.

## Methods

The municipality selected schools for the approach based on their location (i.e., in a deprived neighborhood) or their student population (i.e., a large share of children in vulnerable circumstances, which was determined based on factors such as educational level of the parents). In order for schools to be eligible for the approach, educational quality and internal organization of the schools—as judged by the Dutch Inspectorate of Education—needed to be adequate [12]. Schools needed to create a customized plan, presenting their goals, the chosen initiatives, and a budget plan, at the start of every school year they participated. Based on their experiences and altered circumstances, schools were allowed to deviate from the plan during the course of the year. Policy officers of the domains education, health, and poverty of the municipality were also involved, created the outline of the approach, and helped shape it.

This study was performed in the first four schools participating in the integrated approach, and in one sister school. These schools differed from schools that subsequently participated in the approach in that these schools were in a different city district, participated in the approach at an earlier point in time, and received an additional subsidy from a funding body serving vulnerable groups in society. This subsidy was to be spent on improving children’s health. In these schools, the integrated approach ran during 2 school years from September 2016 to July 2018. A policy officer of the city district coordinated the integrated approach on a local level to facilitate implementation and to strengthen the integrated approach. Principals of the schools got together in monthly meetings with this coordinator to discuss the progress they made, their experiences, and the problems they encountered. At the end of the first school year, one of the schools lost its subsidy for the integrated approach, but remained eligible for the health subsidy. Therefore, all five schools were part of the evaluation study for the entire study period.

The included schools varied in terms of size, student population, ideological basis, and the number, type, and reach of yet implemented initiatives. However, all were relatively small schools in a deprived urban neighborhood that were able to implement or continue several self-chosen initiatives as a result of the integrated approach. In addition, new initiatives of the municipality were implemented in all these schools, such as a weekly consultation hour of a social worker. A more detailed description of the implemented initiatives can be found in the process evaluation of the implementation of the integrated approach [20].

### Study design and data collection

**Fig. 1:**
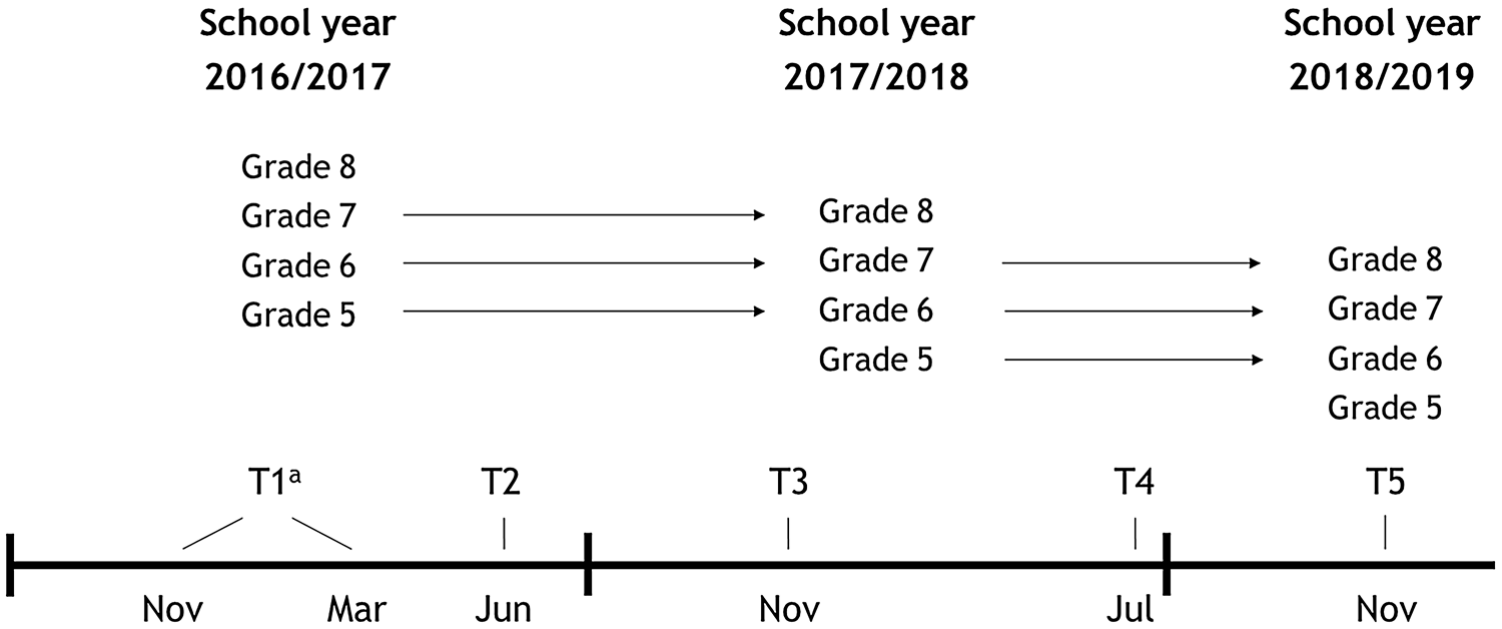
Overview of the design of this study. ^a^At one of the schools, the first measurement was performed in March 2017 and no second measurement was performed

Data collection took place at included schools in four grades (grade 5–8). Children in the included grades were between 7 and 13 years old. Children filled out questionnaires at five measurement occasions over the course of 3 school years. Once at the start of the approach (November 2016), three times during the approach (June 2017, November 2017, and July 2018), and once after the approach was finished (November 2018). The same grades were included at every measurement occasion. Therefore, new children were added to the cohort (lowest grade) and removed from the cohort (highest grade) every school year making the cohort a dynamic cohort (see Fig. 1).

At one of the schools, due to logistical issues, the first measurement (T1) was performed in March 2017 instead of November 2016. The second measurement (T2) in June 2017 was not performed at this school. The remaining measurements (T3–T5) were performed at the same point in time as at the other schools: November 2017 (T3), July 2018 (T4), and November 2018 (T5). Researchers administered the questionnaires in the classroom. During this time, the researchers were available to answer any questions the children might have.

Several weeks before every measurement occasion, parents were informed about the study through an information letter, an announcement in the school newsletter, and flyers at the school entrance. Through these same channels, parents were informed that a researcher would be present at the schools several days before the questionnaires would be administered to provide information and/or answer questions about the study. The parents could inform either the school team or the researchers that they did not want their child to participate in the study by returning the non-consent slip at the bottom of the information letter to their child’s teacher or by emailing the research team. Following withdrawal, children were excluded from all subsequent measurement occasions.

### Measures

Through the questionnaires, information on various measures was collected.

#### Quality of life

Quality of life (QoL) was measured with the KIDSCREEN questionnaire [23]. Parents reviewed the questions and due to their objections, several items were excluded. This concerned items of the KIDSCREEN-52 subscales psychological well-being, moods and emotions, self-perception, parent relation and home life, and financial resources. However, all items of the abbreviated KIDSCREEN-10 and all items of the KIDSCREEN-52 subscales physical well-being (5 items), autonomy (5 items), peers and social support (6 items), school environment (6 items), and bullying (3 items) were included. Response categories to each item were ‘not at all/never’, ‘slightly/seldom’, ‘moderately/sometimes’, ‘very/often’, and ‘completely/always’. A sum score for all subscales with ≥ 5 items was determined when at maximum 1 response was missing. Sum scores of subscales with less than five items were only determined when all items had a valid response. Sum scores were subsequently converted to *T*-scores scaled to scores with mean 50 and standard deviation 10 using scoring algorithms developed by the KIDSCREEN developers. A higher score indicated a higher quality of life. The KIDSCREEN-10 is considered a reliable questionnaire for measuring quality of life in children of age 8–18 years (*α* = 0.82, test–retest ICC = 0.70) and has been shown to have good convergent and criterion validity [24].

#### Psychosocial problems

Children’s psychosocial problems were measured with the Strengths and Difficulties Questionnaire (SDQ). This is a 25-item questionnaire consisting of five subscales, i.e., emotional problems, conduct problems, hyperactivity/inattention, peer relationship problems, and prosocial behavior, with five items each [25]. Response categories for each item were ‘not true’, ‘somewhat true’, and ‘certainly true’ [26]. Scores on each of the five subscales were determined if at minimum three items had a valid response. By summing up the scores on the first four of these five subscales, a total difficulty score was determined with higher scores indicating more problems. The SDQ was only administered to children in grade 7 or 8 (age range: 9–13 years). This was done as concurrent validity and reliability of the Dutch youth self-report SDQ has only been established for 9–15-year-old children (*α* = 0.78, test-rest ICC = 0.87) [26].

#### Covariates

The participants reported their sex, date of birth, country of birth, country of birth of their mother and father, and SES. Date of birth was used to determine age of the participant at every measurement occasion. Country of birth of the child, mother, and father were used to determine ethnic background of the child. When the child or, at minimum, one of the child’s parents was born outside the Netherlands, the participant was classified as having a ‘Western’ (Europe, North-America, Oceania, Japan, and Indonesia) or ‘non-Western’ (all remaining countries) ethnic background. This is the standard classification of Statistics Netherlands [27]. Otherwise, the participant was classified as ‘Dutch’. When participants provided information on their own country of birth only, ethnic background was assumed to be ‘non-Western’ as this was considered most likely in the neighborhood under study. To measure SES, an adapted version of the Family Affluence Scale (FAS) was used asking children about socio-economic indicators of their family and home situation they likely have knowledge on [28–30]. SES was determined for every measurement occasion by adding up the answers to the following questions: (1) do you have a bedroom to yourself (no/yes), (2) does your family own a car (no/yes), (3) in the past year, did you go on holiday outside the Netherlands (no/yes), and (4) which of the following appliances do you have at home (dishwasher/washer/dryer, multiple answers possible). Answers to this last question were coded from ‘1’ to ‘3’ based on whether participants reported having 1, 2, or all 3 of these appliances in their home. Answers to the first three questions were coded ‘0’ (no) and ‘1’ (yes). Sum scores were determined when participants provided a valid answer to at least three of the four questions. The missing response was then assumed to be ‘no’ or ‘0’.

### Statistical analysis

To test whether there were changes in quality of life and in psychosocial problems over the course of the approach, generalized estimating equations (GEE) were performed. This regression method corrects for within-subject correlations by assuming a pre-specified correlation structure for the repeated measures [31]. For both outcome measures, an unstructured correlation structure showed the best fit. Using this correlation structure, no specific correlation structure is imposed on the data and each variance and covariance is separately estimated [31].

Quality of life and psychosocial problems were the dependent variables in two separate GEE models. Time was included as an independent categorical variable in both models. The time variable reflected the five measurement occasions and therefore ranged from ‘1’ for T1 to ‘5’ for T5. The measurements were declared to be at month 0 (T1), 4 (T1), 7 (T2), 12 (T3), 20 (T4), and 24 (T5). The measurements at month ‘0’ and month ‘4’ both belonged to T1 and were therefore combined in the time variable. Sex, age, ethnic background, and SES were included in the models as covariates. As the number of included schools and included grades per school was too low to perform multilevel analyses, school and grade were included in the GEE models as categorical covariates. In addition, as a GEE model with all five time points and psychosocial problems as outcome variable would not converge, T5 was excluded from this analysis, and T1 and T5 were compared in a separate GEE model. All analyses were performed in STATA version 15. To ensure that results cannot be traced back to individual schools, we do not provide separate analyses per school, or sensitivity analyses excluding specific schools.

## Results

Descriptive statistics of the study sample can be found in Table 1. The sample size ranges from 225 to 389 children per measurement occasion. This is estimated to be between 60 and 85% of children in the included grades at the different measurement occasions. In total, 614 children participated in the study. At every measurement occasion, the average age was 10–11 years and the majority (80–83%) of participating children were of non-Western ethnic background. For 5% of the sample, we assumed ethnic background to be non-Western, because only information on own country of birth was provided, and not on either parents’ country of birth.

**Table 1:** Descriptive statistics

	Wave 1		Wave 2		Wave 3		Wave 4		Wave 5	
	Nov 2016/March 2017		Jun 2017		Nov 2017		July 2018		Nov 2018	
	Mean/*n*	SD/%	Mean/*n*	SD/%	Mean/*n*	SD/%	Mean/*n*	SD/%	Mean/*n*	SD/%
*n*^a^	389		225		315		257		255	
Sex										
Girls	212	54.8	132	58.7	167	53.4	131	51.4	125	49.6
Boys	175	45.2	93	41.3	146	46.7	124	48.6	127	50.4
Age (years)	10.04	1.25	10.63	1.23	10.07	1.21	10.64	1.23	10.10	1.30
SES (range 0–6)	3.34	1.25	3.40	1.38	3.55	1.38	3.44	1.38	3.47	1.35
Ethnic background										
Dutch	49	12.7	33	14.7	40	12.9	34	13.4	32	12.7
Western	18	4.7	13	5.8	15	4.8	12	4.7	11	4.4
Non-Western	319	82.6	179	79.6	256	82.3	208	81.9	209	82.9
Grade										
5	95	24.4	50	22.2	74	24.1	71	27.6	64	25.1
6	115	29.6	72	32.0	74	24.1	49	19.1	60	23.5
7	93	23.9	47	20.9	89	29.0	85	33.1	54	21.2
8	86	22.1	56	24.9	70	22.8	52	20.2	77	30.2
SDQ—total (range 0–40)^b^	10.75	5.79	11.29	5.40	10.59	5.58	8.93	5.27	11.33	6.25
KIDSCREEN—10 *T*-score	51.44	10.58	52.35	11.06	53.79	12.73	54.92	11.83	52.24	12.69
KIDSCREEN—52 *T*-scores										
Physical well-being	55.02	10.75	55.19	10.36	56.12	11.75	55.71	10.60	55.64	10.92
Autonomy	49.86	10.98	51.83	10.91	50.66	11.27	53.90	10.19	50.58	12.16
Peers and social support	50.79	10.87	50.85	12.60	51.20	11.22	53.68	11.48	52.15	11.81
School environment	55.70	10.62	51.64	10.55	54.13	10.55	54.69	9.82	52.89	11.10
Bullying	42.56	12.10	44.24	11.01	45.28	11.35	46.65	10.92	43.10	12.46

*SES* socio-economic status, *SDQ* Strengths and Difficulties Questionnaire. Percentages are based on the number of participants per wave with a valid response for that variable

^a^This is the maximum number of participants per wave with a valid response for each of the variables

^b^The SDQ was only administered in children of grade 7 and 8

### Quality of life

The results of the GEE investigating the development of quality of life over the course of the study can be found in Table 2 (*n* = 577). Quality of life was higher at T2 (*B* = 1.68, 95% CI [0.04, 3.31]), T3 (*B* = 2.05, 95% CI [0.66, 3.44]), and T4 (*B* = 3.04, 95% CI [1.24, 4.84]) than at T1. No difference was identified in quality of life between T5 and T1 (*B* = − 0.10, 95% CI [− 1.83, 1.63]).

**Table 2:** Association between time and both health-related quality of life and psychosocial problems tested in generalized estimating equations (GEE) models

	Health-related quality of life^a^			Psychosocial problems^a,b^		
	Coefficient	95% CI		Coefficient	95% CI	
		Lower limit	Upper limit		Lower limit	Upper limit
Time^c^						
1	Ref	Ref	Ref	Ref	Ref	Ref
2	1.68	0.04	3.31	− 0.05	− 1.21	1.12
3	2.05	0.66	3.44	− 0.15	− 1.18	0.87
4	3.04	1.24	4.84	− 1.33	− 2.50	− 0.17
5	− 0.10	− 1.83	1.63	−	−	−
Sex						
Boys	Ref	Ref	Ref	Ref	Ref	Ref
Girls	0.11	− 1.54	1.76	− 0.75	− 1.89	0.39
Age	− 0.28	− 2.08	1.53	− 0.06	− 1.17	1.06
SES	0.40	− 0.12	0.92	− 0.61	− 0.99	− 0.23
Ethnic background						
Dutch	Ref	Ref	Ref	Ref	Ref	Ref
Western	− 4.90	− 9.47	− 0.32	− 0.30	− 3.52	2.93
Non− Western	− 0.03	− 2.44	2.38	− 1.88	− 3.38	− 0.38
Grade						
5	Ref	Ref	Ref	−	−	−
6	2.33	− 0.02	4.69	–	–	–
7	4.88	0.84	8.93	Ref	Ref	Ref
8	4.14	− 1.48	9.76	− 0.64	− 2.09	0.82

In these models, school was additionally included as a categorical covariate, but the school-specific estimates are not shown

SES = socio-economic status; CI = confidence interval

^a^Higher scores indicate higher quality of life and more psychosocial problems

^b^Psychosocial problems were only measured in grade 7 and 8 (age 9–13 years) and it was not possible to include T5 in the GEE models (comparing T5 with T1 separately: *B* = 1.18, 95% CI [− 0.20, 2.56])

^c^Corresponding to T1–T5, with T1 containing the measurement occasions at both 0 and 4 months

**Fig. 2:**
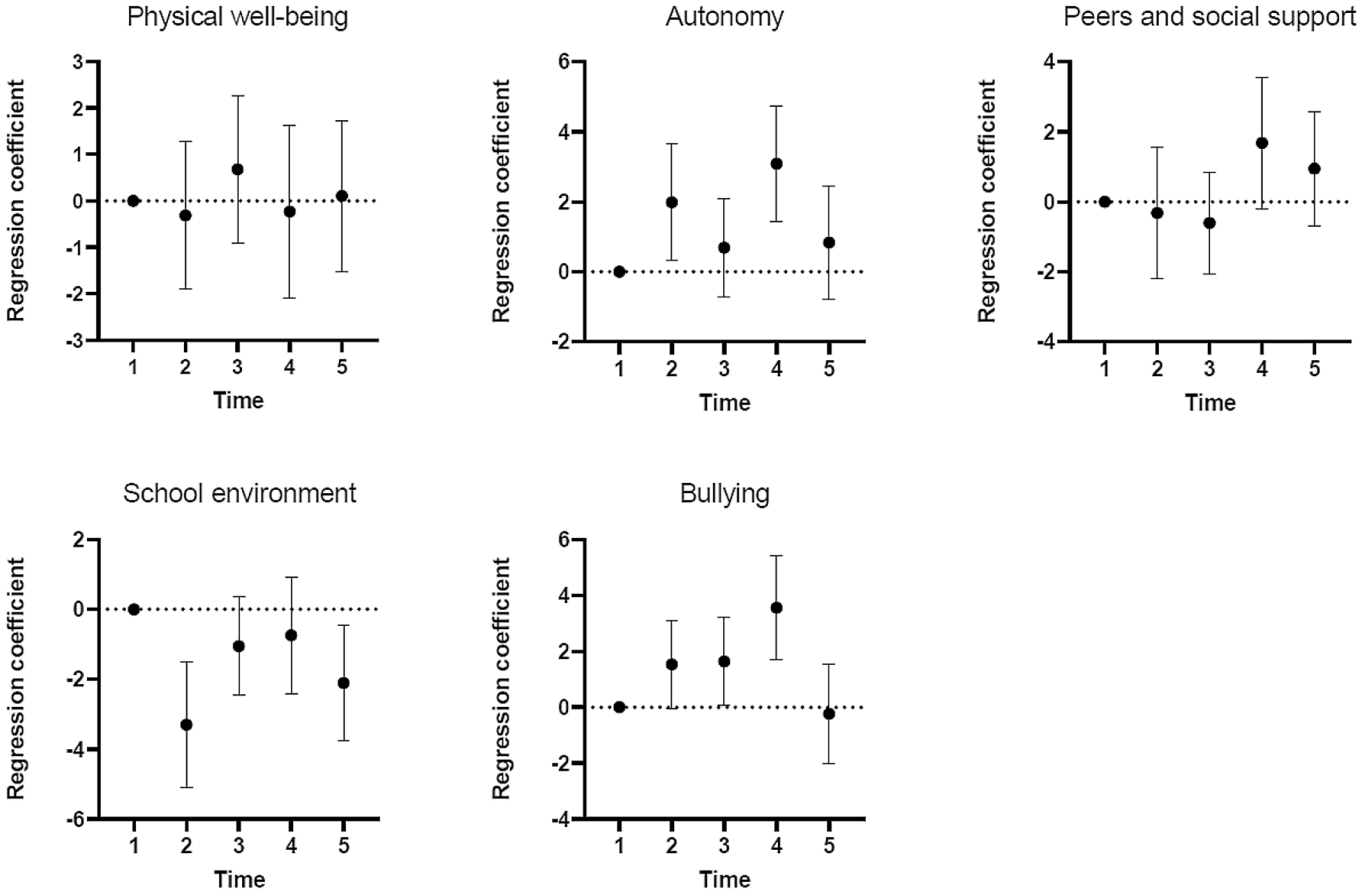
Association between health-related quality-of-life subscales and time tested in generalized estimating equations (GEE) models. These models were adjusted for sex, age, socio-economic status (SES), ethnic background, grade, and school. Time 1–5 corresponds to T1–T5, with T1 containing the measurement occasions at both 0 and 4 months

Figure 2 displays the development in the quality-of-life subscales. No changes were identified from T1 to T5 in scores on the subscales physical well-being (*n* = 588) and peers and social support (*n* = 587). Scores on the autonomy subscale (*n* = 584) were higher, indicating more autonomy, at T2 (*B* = 1.99, 95% CI [0.31, 3.66]) and T4 (*B* = 3.09, 95% CI [1.44, 4.74]) when compared to T1. Scores on the school environment subscale (*n* = 583) were lower, indicating a poorer environment, at T2 (*B* = − 3.30, 95% CI [− 5.10, − 1.50]) and T5 (*B* = − 2.10, 95% CI [− 3.75, − 0.46]) than at T1. Scores on the subscale bullying (*n* = 582) were higher, indicating less bullying, at T2 (*B* = 1.54, 95% CI [− 0.03, 3.10]), T3 (*B* = 1.64, 95% CI [0.07, 3.20]), and T4 (*B* = 3.55, 95% CI [1.70, 5.41]) when compared to T1.

### Psychosocial problems

Table 2 displays the development in psychosocial problems over the first four time points of the study. Psychosocial problems were lower at T4 than at T1 (*n* = 293, *B* = − 1.33, 95% CI [− 2.50, − 0.17]). When comparing T5 with T1 separately, no difference in psychosocial problems between the two time points was identified (*n* = 294, *B* = 1.18, 95% CI [− 0.20, 2.56]).

## Discussion

This study evaluated changes in children’s quality of life and psychosocial problems over the course of a 2-year school-based integrated approach implemented by the municipality in a deprived neighborhood in Amsterdam. Overall, similar patterns for both outcomes were identified with improvements at the end of the approach and scores returning back to baseline after the approach ended. The pattern identified for quality of life more clearly indicated a gradual increase over the course of the approach with mean scores changing from low to within the normal range of Dutch norm data [23]. These identified improvements in quality of life seemed attributable to changes in bullying and autonomy, but not to changes in school environment, physical well-being, and peers and social support. This study shows that an integrated approach, targeted at the interrelated domains of poverty, health, and academic achievement, could contribute to improvements in socio-emotional health of children in a deprived urban neighborhood. As problems in childhood in these domains are interrelated, establishing improvements in health is suggested to be conditional on and a prerequisite for establishing changes in poverty and academic achievement [10, 11, 21, 22]. We also importantly show that these improvements were not sustained. Future research should examine whether implementing integrated approaches structurally can result in sustained improvements in multiple domains, and examine whether this should be done in practice.

In line with our expectations, improvements in quality of life and psychosocial problems were identified at the end of approach. While no control group was incorporated in this study, our process evaluation of the implementation of the integrated approach can guide the interpretation of the results of this study [20]. The process evaluation showed that many initiatives that were implemented at the schools and were regarded successful by school principals targeted children’s socio-emotional health, e.g., hiring a behavioral specialist or implementing positive behavior support and school climate interventions. These types of interventions, focused on social and emotional competencies, have been related to improved behavioral functioning in children [32] making it plausible that the identified changes are related to the implementation of the integrated approach. In addition, an earlier school-based integrated approach showed similar results. Targeting factors such as socio-emotional health and academic development, this approach was shown to be related to improved socio-emotional functioning in children [15, 16]. Likewise, a whole-school approach focusing on educational as well as behavioral aspects was related to improved behavioral functioning [18]. A recent study on a multi-faceted school-based mental health program in urban minority youth, with a focus on the entire school environment and collaboration with multiple agencies and actors, also showed a reduction in socio-emotional problems after the intervention [33]. However, this study did not assess whether the effect was sustained. The current study is the first to show that a school-based integrated approach targeting multiple domains and environments through the involvement of various agencies and actors is related to improvements in children’s socio-emotional health. It also showed that this effect was not sustained after the end of the approach.

There are similarities between the results for quality of life and psychosocial problems, but important differences are to be noted as well. Improvements in quality of life were identified earlier in time and seemed to show a more clear pattern of gradual change over the course of the approach than improvements in psychosocial problems. In addition, where quality of life returned back to baseline after the integrated approach, children almost seemed to experience more psychosocial problems after the approach ended when compared to at the start. The effect estimate of the deterioration after the end of the approach was nearly the same size as the improvement at the end of the approach, but it had a larger variance, and therefore, it was not statistically significant. We expect the deterioration in quality of life and psychosocial problems after the end of the approach to be due to initiatives coming to an end. It suggests that approaches need to have a more structural character in order for improvements to be sustained. The fact that we even nearly identified an increase in psychosocial problems after the end of the approach compared to at the start could hint at a potential harmful effect of the ending of the approach. This may suggest that interventions need to be ended more gradually, or that children need to be better prepared for interventions ending. At the very least we think that, when multi-faceted interventions come to an end, different components can probably best be ended at different points in time.

The differences between the results of the quality of life and psychosocial problem measures can be caused by the fact that they measure a different concept. Psychosocial problems reflect behavioral and emotional problems. Quality of life is an inversely related, but slightly wider, concept [3] covering physical, emotional, mental, social, and behavioral dimensions of well-being and functioning [24]. Possibly, the integrated approach has a bigger impact on children’s overall daily functioning than on their psychosocial problems. Besides differences between the concepts, differences in the results can be caused by the fact that the two measures were studied in different samples. Quality of life was measured in the full sample, while psychosocial problems were only measured in children over or equal to the age of 9 years. It is possible that changes in younger children were more pronounced than changes in older children. Children in the lower grades did seem to generally score lower on quality of life than children in the higher grades, with more pronounced differences at baseline than over the course of the approach. In addition, restricting the sample for the psychosocial problems measure caused the sample size to be lower which could have resulted in too low power to detect differences on this measure. However, the observed effect estimates at T2 and T3 compared to T1 are around zero. This is indicative of a lack of effect, rather than a lack of power.

When comparing the results of the quality of life subscales to those of the overall quality of life scale, the subscales bullying and autonomy showed a similar pattern as overall quality of life. On these subscales, the mean scores seemed to change from low to within the normal range of Dutch norm data [23]. The other subscales showed different patterns. For the subscale bullying, this result is expected as it is a measure reflective of school climate, which was targeted by multiple implemented initiatives that were considered to be successful by schools. In addition, school-wide positive behavioral support interventions have been shown to be related to lower rates of bullying and peer rejection in earlier research, presumably via improvements in school climate [34]. For the subscale autonomy, the pattern only deviated from the pattern of overall quality of life in that at the beginning of the second year of the approach the score dropped back to baseline. Changes in autonomy were not anticipated upon, as initiatives did not specifically seem to target autonomy. Autonomy is a measure reflecting children’s feeling of having control over how they spend their time. The specific pattern of improvement could be due to a learning effect as improvements were only identified at the two second measurement occasions within a school year (T2 and T4). However, the nature of the measure and the fact that we only observed this pattern for this subscale make this explanation unlikely.

No or only few initiatives targeted physical well-being and peers and social support [20]. Therefore, it was not unexpected that no differences were identified in these subscales over the course of the approach. For the subscale school environment, deteriorations were seen. Scores for school environment were lower at the end of the first year of the integrated approach and after the integrated approach ended than they were at baseline. This seems counterintuitive, as implemented initiatives targeting school climate were regarded successful. However, the subscale school environment measures children’s own functioning within the school rather than the school climate, which could explain this result. Nevertheless, compared to at the start of the approach, children seemed to enjoy school less or to function less well in the school or class at the end of the first year of the integrated approach and after the integrated approach ended. From our process evaluation, we know that teacher shortage and other issues at the schools at times could cause instability in the school environment for children, which could potentially explain these results [20]. We have so far focused on theoretical explanations for the results. However, it is important to note that positive changes were mainly established in domains in which the children at baseline scored relatively lower, differences with the Dutch norm data were relatively bigger, and the mean scores could be considered low compared to the norm data [23]. Although we do not think that we encountered a ceiling effect for the subscales where children scored higher at baseline, changes on subscales where children at baseline scored lower may have been more likely to occur and be identified.

### Strengths and limitations

The current study is unique in that it uses data on children’s socio-emotional health from five measurement occasions over the course of and after a 2-year school-based integrated approach implemented by the municipality in a deprived neighborhood in Amsterdam. Another strength of this study is that it uses two different measures that reflect two different aspects of children’s socio-emotional health, i.e., quality of life and psychosocial problems. Finally, the fact that a process evaluation was performed and used to guide the interpretation of the results of this study is a particular strength.

The fact that this study evaluated changes in children’s socio-emotional health in schools in which a municipal integrated approach was implemented was a strength of this study, but it also carried limitations. First, it meant that we could not include a control group as there were no suitable schools to include in the control group. Second, the number of children we were able to include may have been too low to identify changes in psychosocial problems, and also too low for a model testing changes in psychosocial problems including all measurement occasions to converge. However, as all children in the final grades of the schools involved in the integrated approach were approached to participate in the study, there was no way to overcome this limitation. In addition, due to the small number of included schools and classes, we were unable to take into account the nesting of students in schools and grades by applying multilevel models. However, we did incorporate school and grade as covariates in the GEE.

Also, due to logistical issues, the first measurement occasion at one of the schools was approximately 4 months later than at the other schools and the second measurement occasion was skipped at this school. We expect that this has potentially resulted in an underestimation of the evaluated changes, as the baseline measurement for this school was done several months after the start of the integrated approach. Another limitation of this study is that while we identified statistically significant changes in children’s psychosocial problems and quality of life, with mean scores going from low to within the normal range, differences were generally small. It is therefore unclear whether the identified improvements can also be considered relevant. A final limitation is that we do not know the exact response rate of our study, as we do not know exactly how many children were in the included grades of the participating schools.

## Conclusion

This study is unique in that it evaluated changes in children’s quality of life and psychosocial problems over the course of and following a school-based integrated approach targeting multiple domains and environments through the involvement of various agencies and actors. In schools in a deprived urban neighborhood, improvements in both children’s quality of life and psychosocial problems were identified at the end of the approach and were shown to be followed by returns back to baseline after the approach ended. The integrated approach is likely to underlie these changes as many implemented initiatives, regarded as successful by school principals, targeted children’s socio-emotional health. This study shows that integrated approaches, including a wide range of initiatives focusing on education, health, and poverty, could contribute to improvements in children’s socio-emotional health. However, the results also imply that these improvements are domain-specific. Moreover, the results importantly suggest that these approaches may need to be implemented structurally, or at least longer-term, in order for established improvements to be sustained.

## Data Availability

Not applicable.
